# Micronutrient Intakes among Children and Adults in Greece: The Role of Age, Sex and Socio-Economic Status

**DOI:** 10.3390/nu6104073

**Published:** 2014-10-03

**Authors:** Yannis Manios, George Moschonis, Christina Mavrogianni, Rolf Bos, Cécile Singh-Povel

**Affiliations:** 1 Department of Nutrition and Dietetics, Harokopio University of Athens, 70 El Venizelou Avenue, Kallithea, 17671 Athens, Greece; E-Mails: gmoschi@hua.gr (G.M.); cmavrog@hua.gr (C.M.); 2 EnviNHealth S.A., Vasilissis Sofias 22, Marousi, 15124 Athens, Greece; 3 FrieslandCampina, Stationsplein 4, Post Box 1551, Amersfoort 3800 BN, The Netherlands; E-Mails: rolf.bos@frieslandcampina.com (R.B.); Cecile.Povel@frieslandcampina.com (C.S.-P.)

**Keywords:** diet, nutrients, age, socio-economic status, Greece

## Abstract

The aim of the present study was to report the usual nutrient intakes of sixteen micronutrients by schoolchildren, adults and the elderly in Greece and to further explore the role of age, sex and socio-economic status (SES) on meeting the recommended nutrient intakes. Dietary intake, demographic and SES data from three existing studies conducted in Greece (in 9–13-year-old children; 40–60-year-old adults; and 50–75-year-old women) were collected. The prevalence of study participants with inadequate micronutrient intakes were assessed using the estimated average requirement (EAR) cut-point method. Regarding sex and age differences, the highest prevalences of inadequate nutrient intakes occurred in post-menopausal women. In both sexes and all age groups, the prevalence of vitamin D intake below EAR reached 100%. Furthermore, nutrient intakes of 75% or more below EAR were found for vitamin E in all age groups, folate in women and for calcium and magnesium in post-menopausal women (*p* < 0.05). Regarding SES differences, the prevalences of inadequate calcium and vitamin C intakes were higher for children and postmenopausal women of lower SES compared to their higher SES counterparts (*p* < 0.05). The current study reported the highest prevalences of inadequate intakes for both sexes and all age and SES groups for calcium, folate and vitamins D and E. These findings could provide guidance to public health policy makers in terms of updating current dietary guidelines and fortifying foods to meet the needs of all population subgroups.

## 1. Introduction

Lifestyle changes occurring over the last few decades in many developed countries, including Greece, are characterized by increased consumption of low-cost, but energy-dense foods and by reduced physical activity levels. These lifestyle changes are the major factors implicated in the etiology of the current obesity epidemic [1]. However, even with excess dietary intake of energy and macronutrients, it is still questionable whether the population is meeting the recommended intakes of micronutrients. In this context, a number of national epidemiological surveys conducted in several developed countries have reported the coexistence of obesity with inadequate intakes for certain vitamins and minerals, notably calcium, iron, zinc, vitamins B_1_, B_2_, B_6_, D and folate [2,3,4,5]. Still, the findings on the prevalence of inadequate micronutrient intakes have not been consistent among studies conducted in different developed countries. Regarding Greece in particular, the relevant data for both children and adults are either old or scarce, thus requiring further investigation.

Further with regard to the above, any public health initiative aiming to ensure adequate micronutrient intakes of the population should be based on accumulative evidence-based findings on any potential insufficient dietary intakes. This approach has been very efficiently followed by several studies, such as the National Health and Nutrition Examination Survey (NHANES) [6] in the United States, where inadequate nutrient intakes have been thoroughly recorded, not only for the overall population, but most importantly, for specific age and societal groups. Therefore, recording and understanding the potential insufficient dietary intakes of the population, as well as identifying the specific population subgroups most in need for any such public health initiatives seems the most promising approach in guiding tailored-made strategies to most effectively tackle this public health issue.

The aim of the current study was to report the usual nutrient intakes of 16 micronutrients using data from existing studies conducted in Greece on schoolchildren, adults and the elderly. Furthermore, the role of age and socio-economic status on meeting the recommended dietary intakes for each one of these 16 micronutrients was further explored.

## 2. Subjects and Methods

### 2.1. Participants

Data from existing studies conducted in Greece on schoolchildren, adults and the elderly were used in the current work. All studies were conducted according to the guidelines laid down in the Declaration of Helsinki, and all procedures involving human subjects were approved by the Ethics Committee of Harokopio University (Ethics approval code for the Healthy Growth Study: 16/19-12-2006; Ethics approval code for both the CardioHealth Study and the Postmenopausal Health Study II: 18/01-11-2007). Written informed consent was obtained from all study participants.

#### 2.1.1. Healthy Growth Study

The “Healthy Growth Study” (HGS) was a cross-sectional epidemiological study initiated in May, 2007. In addition to the ethical approval, approval to conduct the study was also granted by the Greek Ministry of National Education. The study population comprised schoolchildren (9–13 years old) attending the fifth and sixth grades of primary schools located in municipalities within the wider regions of four Greek counties. The sampling procedure is described in detail elsewhere [7]. In brief, the sampling of schools was random, multistage and stratified by parental educational level and by the total population of students attending schools within these municipalities. An extended letter explaining the aims of the present study and a consent form for conducting full measurements were provided to all parents or guardians having a child in these schools. Signed parental consent forms were collected for 2660 out of 4145 children (response rate 64%). The current analysis included a representative sample of 1100 children with full dietary intake and socio-demographic data.

#### 2.1.2. CardioHealth Study

In 2007, an open invitation for participation in the study was advertised, and a total of 340 men and women volunteered to participate from the wider district of Athens. Based on the eligibility criteria used for the participation of volunteers in the current study, presented elsewhere [8], 154 middle-aged men and women (40–60 years old) were considered eligible, and full dietary intake and socio-demographic data were collected.

#### 2.1.3. Postmenopausal Health Study II

In March, 2008, volunteers were invited to participate by informational brochures and posters distributed in public buildings and community centers in municipalities from the wider district of Athens. The study protocol registration number was NTR1396. Through the initial screening of the study, a sample of 720 postmenopausal women volunteered to participate. Based on the eligibility criteria used for the participation of volunteers in the current study, described elsewhere [9], 214 women (50–75 years old) were identified and were invited to participate in the study, and full dietary intake and socio-demographic data were collected from them.

### 2.2. Dietary Intake

Dietary intake data were obtained by trained dieticians and nutritionists via morning interviews with children at the school site (in the case of HGS) and with adult study participants during scheduled meetings at Harokopio University in the case of the CardioHealth study and the Postmenopausal Health Study (PHS) II. Dietary intake data were collected for two consecutive weekdays and one weekend day in all three studies, using the 24-h recall technique. All interviewers were rigorously trained to minimize interviewer effects. Study participants were asked to describe the type and amount of food, as well as all the beverages consumed during the previous day, provided that it was a usual day according to the participant’s perception. To ensure quality, food models and sample household measurements (such as cups and spoons) were used to specify serving sizes. At the end of each interview, the interviewers reviewed the collected food intake data with the respondent in order to clarify entries, servings and possible forgotten foods. After completing the 24-h recall, subjects were asked whether they were using dietary supplements, and when the reply was positive, they were further asked about the type of supplement, the dosage and the frequency of intake. This information was used when assessing total dietary intake, which included the intake of the extra nutrients provided by supplements. Furthermore, information on the brands of all packaged foods consumed by study participants was also recorded during the 24-h recalls and was used to identify if any of these foods were fortified. A trained dietician checked the food records for any misrecorded or missing information, while a random 10% of all records was re-entered in the food database by a different person than the one entering the original data. The data were then compared with the original to ensure inter-researcher reliability. Food intake data were analyzed with Nutritionist V diet analysis software (version 2.1, 1999, First Databank, San Bruno, CA, USA), which was extensively amended to include traditional Greek foods and recipes, as described in the Food Composition Tables and Composition of Greek Cooked Food and Dishes [10,11]. Furthermore, the databank was updated with nutrition information of chemically analyzed commercial food items widely consumed by children and adults in Greece, including fortified foods.

The distribution of usual intakes of 16 vitamins and minerals was estimated by using the National Research Council method (NRC method), which attempts to remove the effects of day-to-day (within subject) and subject-by-subject (between-subject) variability in dietary intakes [12]. More specifically, the following equation was used for the calculation of the adjusted (usual) dietary intake:

[(subject’s mean − group mean) × SD_between-person_/SD_observed_] + group mean (1)

To check for underreporting, the ratio of reported energy intake (EI) and the predicted basal metabolic rate was used. The basal metabolic rate (BMR) was estimated according to Schofield equations [13], taking into account age, sex and body weight. For the identification of those study participants that under-reported their energy intake, the age and sex-specific EI:BMR ratio cut-off points proposed by Goldberg *et al.* [14] were used. This is a population-based method that takes into account the size of the sample population and the number of days of dietary intake records for the detection of under-reporters. The identified under-reporters were not included in the analyses conducted in the current study. More specifically, in the case of the HGS, the subsample of 1100 children examined in the current analyses did not include under-reporters, while correct reporting of energy intake was one of the main eligibility criteria for participation in the two adult studies.

The estimated average requirement (EAR) cut-point method proposed by IOM (Institute of Medicine) was used to assess the adequacy of the micronutrient intakes of children, adults and elderly women [15,16]. In summary, EAR is the average daily nutrient intake level estimated to meet the nutrient requirements of 50% of all healthy individuals within a group. The EAR cut-point method is a statistical method of determining the prevalence of nutrient inadequacy in a group, assessing the proportion of individuals in the group whose usual nutrient intakes are below the EAR [15].The rational of using IOM’s dietary intake reference values was based on the fact that these are regularly updated and frequently used compared to reference values provided by other scientific bodies of organizations. For micronutrients with an EAR, the EAR threshold was used to estimate the proportion of each one of the three populations with usual intakes below the EAR [15,17]. For only one micronutrient (*i.e*., potassium), for which there are no established EAR values, the proportion below the adequate intake (AI) threshold was used alternatively. The EAR cut-off points for 15 of the micronutrients under study and the AI for potassium are summarized in Supplementary Table 1. In order to define the inadequacy of nutrient intake by the populations under study, a threshold of 20% was set with respect to the percentage of study participants having intakes below the EAR values. The rationale for using this specific threshold was based on the available literature reporting inadequate nutrient intakes for adults in Europe [18].

### 2.3. Socio-Economic and Demographic Variables

Data on the socio-economic and demographic characteristics of the three populations under study were collected via interviews with the parents of children in the case of the HGS and study participants in the case of the CardioHealth study and the PHS II. All interviews were conducted by research team members that were rigorously trained to minimize the interviewer’s effect with the use of standardized questionnaires. More specifically, the socio-demographic data collected by parents in the HGS included parents’ age, educational level (years of education), nationality (*i.e.*, country of origin), employment and marital status. Information on children’s date of birth was also collected by parents and was used for the calculation of children’s age. Similarly, the socio-demographic data collected from middle-aged adults and postmenopausal women participating to the CardioHealth study and the PHS II, respectively, included age, educational level (in years) and nationality (*i.e.*, country of origin). Furthermore, data on employment status were also collected from the adults participating in the CardioHealth study.

### 2.4. Statistical Analysis

Continuous variables are expressed as the means (standard deviations, SD), whereas categorical variables are expressed as percentages (%). The Kolmogorov-Smirnov test was used to determine the normality of the distribution of the examined variables. For the comparison of means between groups, one-way analysis of variance was used, using the Bonferroni rule to correct for the inflation in type 1 error due to multiple *post hoc* comparisons. The Chi-square test was used to explore the association between categorical variables, using the two-sample *Z*-test for proportions for multiple *post hoc* comparisons. All *p*-values reported are two-tailed. Statistical analysis was done with SPSS version 21.0. The level of statistical significance was set at *p* < 0.05.

## 3. Results

Table 1 presents certain descriptive socio-economic and demographic characteristics of 9–10- and 11–13-year-old girls and boys (and their parents) participating in the HGS; 40–60-year-old women and men participating in the CardioHealth study; and 50–75-year-old postmenopausal women participating in the PHS II. The average years of education ranged from 10.3 years in 50–75-year-old women to 13.5 years in 40–60-year-old men. Regarding nationality, the vast majority (*i.e.*, 93% to 100%) of adult study participants were nationals, while the relevant percentage was lower for children, ranging from 75.4% to 86%.

**Table 1:** Descriptive socio-demographic characteristics of children (and their parents), middle-aged adults and postmenopausal women.

Socio-Demographic Characteristics	Children				Middle-Aged Adults		Postmenopausal Women
	9–10 Years old		11–13 Years Old		40–60 Years Old		50–75 Years Old
	Boys (*N* = 264)	Girls (*N* = 260)	Boys (*N* = 277)	Girls (*N* = 299)	Men (*N* = 79)	Women (*N* = 75)	(*N* = 214)
	Mean (SD)	Mean (SD)	Mean (SD)	Mean (SD)	Mean (SD)	Mean (SD)	Mean (SD)
Age (years)	(*Children*) 10.5 (0.30)	(*Children*) 10.5 (0.32)	(*Children*) 11.6 (0.47)	(*Children*) 11.6 (0.46)	46.7 (8.15)	50.2 (9.07) *	62.0 (5.62)
	(*Father*) 43.9 (5.46)	(*Father*) 44.5 (5.25)	(*Father*) 44.4 (5.64)	(*Father*) 43.8 (5.51)			
	(*Mother*) 39.5 (4.82)	(*Mother*) 40.2 (5.07)	(*Mother*) 40.4 (5.15)	(*Mother*) 39.6 (5.14)			
Education (years)	(*Father*) 13.3 (3.52)	(*Father*) 12.6 (3.55)	(*Father*) 12.2 (3.68)	(*Father*) 12.4 (3.49)	13.5 (4.02)	12.9 (3.77)	10.3 (4.03)
	(*Mother*) 13.1 (3.11)	(*Mother*) 12.7 (3.13)	(*Mother*) 12.5 (3.18)	(*Mother*) 12.4 (3.23)			
*Nationality (%)*							
Non-nationals	14.0	17.4	18.7	24.6	0.0	7.0 *	0.9
Nationals	86.0	82.6	81.3	75.4	100.0	93.0 *	99.1

* Comparisons between sexes were tested using the Student’s *t*-test (or the non-parametric Mann**-**Whitney test whenever appropriate) in the case of continuous variables, and the Chi-square test (with the two-sample *Z*-test for proportions whenever appropriate) in the case of categorical variables. No statistically significant differences were observed between males and females.

Table 2 and Table 3 summarize the average usual dietary intakes of energy, macronutrients, vitamins and minerals by males and females, respectively, and the differences among age groups. In males, the usual dietary intakes of energy, carbohydrates, protein, total fat, calcium and vitamins B_2_, B_6_ and D were found to decrease with age, being lower in 40–60-year-old men compared to 9–13-year-old boys (*p* ≤ 0.003), while an opposite trend was observed for copper, magnesium, selenium and vitamin E (*p* ≤ 0.003). In females, the usual dietary intake of energy, carbohydrates, protein, total fat, calcium, iron and vitamins B_1_, B_2_, B_12_, B_6_, D and folate were found to decrease with age, being higher in 9–13-year-old girls compared to 40–75-year-old women (*p* < 0.001). In contrast, the usual dietary intakes of magnesium, selenium and vitamin E were found to increase with age, being higher in 40–75-year-old women compared to 9–13 year-old girls (*p* ≤ 0.001).

Table 4 displays the percentages of males and females in the different age groups with inadequate nutrient intakes, as well as the comparisons of these percentages among age groups. In males, the percentages of study participants with inadequate nutrient intakes were found to increase with age for copper, iron, magnesium, zinc and vitamins A, B_1_, B_2_, B_6_, B_12_ and C (*p* < 0.003), while the percentage of males with usual dietary selenium intake below EAR was found to decrease with age (*p <* 0.001). In females, the percentages of study participants with inadequate nutrient intakes were found to increase with age for calcium, magnesium, zinc, vitamins B_1_, B_2_, B_6_, B_12_ and folate (*p* < 0.001). The highest inadequate nutrient intakes occurred in post-menopausal women. In both sexes and all age groups, the percentage of study participants with a usual dietary intake of vitamin D below EAR reached 100%. Furthermore, nutrient intakes of 75% or more below EAR were found for vitamin E in all age groups, folate in women and for calcium and magnesium in post-menopausal women.

Table 5 presents the percentages of study participants in different age groups and different levels of education (parental in the case of children) with inadequate intakes in the examined micronutrients, as well as the comparisons of these percentages among educational level groups. The percentages of inadequate calcium intakes were found to be higher for 9–10 and 11–13 year-old children, whose parents had ≤9 years of education compared to those whose parents had >14 years of education (*p* = 0.003 and *p* = 0.036, respectively). Regarding iron, the percentage of inadequate intake was found to be higher for 11–13 year-old children whose parents had ≤9 years of education compared to those whose parents had 9–14 or >14 years of education (*p* = 0.006). Furthermore, the percentage of inadequate magnesium intake was found to be higher for 40–60 year-old adults with ≤9 years of education compared to those with 9–14 and >14 years of education, respectively (*p* = 0.002). Regarding vitamin A, the percentage of 9–10-year-old children with inadequate intake was found to be higher for children whose parents had ≤9 years of education compared to their peers whose parents had >14 years of education (*p* = 0.013). Regarding B complex vitamins, a higher percentage of inadequate intake was found for vitamin B_2_ in 9–10 year-old children whose parents had ≤9 years of education compared to those whose parents had 9–14 or >14 years of education (*p* = 0.002), while the percentage of inadequate intake for vitamin B_6_ was found to be higher in 9–10-year-old children whose parents had ≤9 years of education compared to their peers whose parents had 9–14 years of education (*p* = 0.006). Lastly, the percentages of inadequate vitamin C intakes were found to be higher for 9–10-year-old children whose parents had <9 years of education and for 50–75-year-old postmenopausal women with ≤9 years of education compared to their counterparts with 9–14 years of education (*p* *=* 0.003 and *p* *=* 0.015, respectively).

**Table 2:** Average energy, macronutrient and micronutrient intakes in boys and middle-aged men in Greece presented by age group.

Dietary Intake	Males			
	9–10 Years Old (*N* = 264)	11–13 Years Old (*N* = 277)	40–60 Years Old (*N* = 79)	*p-*Value ^†^
	Mean (SD)	Mean (SD)	Mean (SD)	
*Energy and macronutrients*				
Energy (kcal/day)	2144.3 (548.0) ^b^	2134.0 (453.4) ^c^	1774.0 (458.6) ^bc^	<0.001
Carbohydrates (g/day)	240.2 (72.6) ^b^	241.1 (63.4) ^c^	182.0 (70.6) ^bc^	<0.001
Fat (g/day)	100.1 (31.6) ^b^	97.8 (28.4) ^c^	78.8 (27.3) ^bc^	<0.001
Protein (g/day)	82.0 (24.1) ^b^	83.6 (24.1) ^c^	71.9 (20.7) ^bc^	0.003
*Micronutrients*				
Calcium (mg/day)	1121.3 (331.7) ^b^	1151.0 (365.5) ^c^	882.4 (362.2) ^bc^	<0.001
Copper (μg/day)	999.4 (581.8) ^b^	1027.6 (515.3) ^c^	1317.8 (1048.9) ^bc^	0.001
Iron (mg/day)	11.7 (3.79)	11.9 (3.93)	12.5 (6.20)	0.381
Magnesium (mg/day)	249.2 (67.5) ^b^	249.4 (62.2) ^c^	285.5 (111.1) ^bc^	0.001
Potassium (g/day)	2.66 (0.75)	2.66 (0.71)	2.46 (0.93)	0.148
Selenium (μg/day)	58.8 (140.4) ^ab^	38.4 (20.9) ^ac^	113.8 (52.3) ^bc^	<0.001
Zinc (mg/day)	11.8 (15.9)	11.3 (3.05)	9.83 (5.14)	0.404
Vitamin A (μg/day)	751.4 (655.5)	908.0 (1550.1)	970.2 (962.9)	0.208
Vitamin B_1_(mg/day)	1.40 (0.49)	1.34 (0.50)	1.25 (0.67)	0.113
Vitamin B_2_ (mg/day)	1.85 (0.54) ^b^	1.92 (0.73) ^c^	1.55 (0.87) ^bc^	0.001
Vitamin B_6_ (mg/day)	1.86 (0.60) ^b^	1.84 (0.62) ^c^	1.56 (0.73) ^bc^	0.003
Vitamin B_12_ (μg/day)	4.84 (2.17)	6.0 (10.2)	4.73 (7.21)	0.151
Folate (μg/day)	246.8 (109.2)	253.6 (106.5)	284.0 (169.3)	0.088
Vitamin C (mg/day)	99.2 (62.7)	100.2 (70.9)	109.1 (93.7)	0.649
Vitamin D (μg/day)	1.72 (1.04) ^b^	1.73 (1.11) ^c^	1.05 (1.17) ^bc^	<0.001
Vitamin E (mg/day)	8.16 (3.02) ^b^	7.76 (3.13) ^c^	9.46 (6.24) ^bc^	0.003

All micronutrient intakes are adjusted for within-subject and between subject variations by the National Research Council (NRC) method. ^†^ Derived from ANOVA. Superscript letters denote significant differences (*p* < 0.05) of mean values between age groups after *post hoc* multiple comparisons using the Bonferroni rule. Figures sharing the same superscript letters differ significantly from each other: ^a^ 9–10 years old *vs.* 11–13 years old; ^b^ 9–10 years old *vs.* 40–60 years old; ^c^ 11–13 years old *vs.* 40–60 years old.

**Table 3:** Average energy, macronutrient and micronutrient intakes in girls, middle-aged women and postmenopausal women in Greece presented by age group.

Dietary Intake	Females				
	9–10 Years Old (*N* = 260)	11–13 Years Old (*N* = 299)	40–60 Years Old (*N* = 75)	50–75 Years Old (*N* = 214)	*p-*Value ^†^
	Mean (SD)	Mean (SD)	Mean (SD)	Mean (SD)	
*Energy and macronutrients*					
Energy (kcal/day)	1834.1 (471.5) ^abd^	1943.8 (498.7) ^ace^	1422.0 (406.7) ^bc^	1555.0 (234.4) ^de^	<0.001
Carbohydrate (g/day)	208.5 (64.3) ^abd^	224.9 (70.8) ^ace^	155.0 (49.2) ^bc^	171.7 (35.9) ^de^	<0.001
Fat (g/day)	84.3 (26.6) ^bd^	88.6 (28.2) ^ce^	65.5 (25.8) ^bc^	72.5 (15.8) ^de^	<0.001
Protein (g/day)	70.3 (21.9) ^bd^	72.5 (24.2) ^ce^	57.6 (17.7) ^bc^	61.8 (14.9) ^de^	<0.001
*Micronutrients*					
Calcium (mg/day)	1003.9 (313.5) ^bd^	1040.0 (350.5) ^ce^	738.7 (367.1) ^bc^	760.1 (244.1) ^de^	<0.001
Copper (μg/day)	975.8 (1368.1)	926.2 (318.2)	991.5 (496.7)	1030.1 (358.7)	0.623
Iron (mg/day)	10.4 (4.2) ^d^	11.7 (6.6)^ ce^	8.77 (3.08) ^c^	9.31 (2.38) ^de^	<0.001
Magnesium (mg/day)	225.7 (56.3) ^b^	232.9 (64.9) ^c^	262.6 (159.9) ^bcf^	225.4 (51.8) ^f^	0.003
Potassium (g/day)	2.39 (0.67)	2.43 (0.77)	2.49 (1.33)	2.34 (0.64)	0.411
Selenium (μg/day)	58.9 (187.1) ^bd^	48.7 (83.5) ^ce^	176.5 (406.7) ^bc^	173.2 (204.1) ^de^	<0.001
Zinc (mg/day)	11.7 (34.6)	10.1 (3.85)	7.17 (2.98)	7.28 (7.16)	0.086
Vitamin A (μg/day)	712.5 (527.5) ^b^	695.0 (483.2) ^c^	1085.3 (2474.2) ^bcf^	752.1 (239.3) ^f^	0.016
Vitamin B_1_ (mg/day)	1.22 (0.51) ^bd^	1.20 (0.50) ^ce^	0.98 (0.42) ^bc^	0.90 (0.24) ^de^	<0.001
Vitamin B_2_ (mg/day)	1.66 (0.56) ^bd^	1.72 (0.61) ^ce^	1.24 (0.46) ^bc^	1.23 (0.35) ^de^	<0.001
Vitamin B_6_ (mg/day)	1.62 (0.55) ^bd^	1.71 (0.66) ^ce^	1.27 (0.45) ^bc^	1.35 (0.44) ^de^	<0.001
Vitamin B_12_ (μg/day)	4.25 (2.05)	4.38 (2.58) ^c^	2.48 (1.15) ^c^	3.86 (6.98)	0.015
Folate (μg/day)	226.9 (106.9) ^d^	237.0 (155.06) ^e^	227.9 (97.3)	216.8 (46.8) ^de^	0.001
Vitamin C (mg/day)	97.0 (66.7)	94.2 (66.1)	111.5 (82.5)	96.6 (49.6)	0.390
Vitamin D (μg/day)	1.56 (0.91) ^d^	1.54 (1.03) ^e^	1.39 (1.14) ^f^	0.91 (0.57) ^def^	<0.001
Vitamin E (mg/day)	7.40 (3.26) ^d^	7.79 (3.99) ^e^	7.58 (3.62)	9.01 (2.40) ^de^	0.001

All micronutrient intakes are adjusted for within-subject and between subject variations by the NRC method. ^†^ Derived from ANOVA. Superscript letters denote significant differences (*p* < 0.05) of mean values between age groups after *post hoc* multiple comparisons using the Bonferroni rule. Figures sharing the same superscript letters differ significantly from each other: ^a^ 9–10 years old *vs.* 11–13 years old; ^b^ 9–10 years old *vs.* 40–60 years old; ^c^ 11–13 years old *vs.* 40–60 years old; ^d^ 9–10 years old *vs.* 50–75 years old; ^e^ 11–13 years old *vs.* 50–75 years old; ^f^ 40–60 years old *vs.* 50–75 years old.

**Table 4:** Percentages of children, middle-aged adults and postmenopausal women in Greece with usual dietary intakes below estimated average requirement (EAR), presented by sex and age group.

Micronutrients	Males				Females				
	9–10 Years Old (*N* = 264)	11–13 Years Old (*N* = 277)	40–60 Years Old (*N* = 79)	*p*-Value ^‡^	9–10 Years Old (*N* = 260)	11–13 Years Old (*N* = 299)	40–60 Years Old (*N* = 75)	50–75 Years Old (*N* = 214)	*p*-Value ^‡^
	%	%	%		%	%	%	%	
Calcium (mg/day)	53.0	49.1	41.7	0.199	64.6 ^d^	61.2 ^e^	62.5 ^f^	88.3 ^def^	<0.001
Copper (μg/day)	1.9 ^b^	2.9 ^c^	18.3 ^bc^	<0.001	7.7 ^b^	8.4 ^c^	37.5 ^bcf^	1.9 ^f^	<0.001
Iron (mg/day)	1.1 ^b^	0.7 ^c^	6.7 ^bc^	0.002	1.9 ^b^	2.3 ^c^	29.2 ^bcf^	1.9 ^f^	<0.001
Magnesium (mg/day)	24.6 ^b^	23.5 ^c^	71.7 ^bc^	<0.001	38.1 ^bd^	35.5 ^ce^	64.6 ^bc^	80.7 ^de^	<0.001
Potassium (g/day)	97.3	98.2	98.3	0.773	98.8	99.0	93.8 ^f^	99.5 ^f^	0.010
Selenium (μg/day)	50.0 ^b^	57.8 ^c^	8.3 ^bc^	<0.001	58.8 ^bd^	57.9 ^ce^	10.4 ^bc^	5.2 ^de^	<0.001
Zinc (mg/day)	4.2 ^b^	6.9 ^c^	53.3 ^bc^	<0.001	13.5 ^bd^	13.7 ^ce^	50.0 ^bc^	59.9 ^de^	<0.001
Vitamin A (μg/day)	24.2 ^b^	26.0 ^c^	46.7 ^bc^	0.003	26.9 ^d^	22.7 ^e^	39.6 ^f^	9.8 ^def^	<0.001
Vitamin B_1_ (mg/day)	4.2 ^b^	0.7 ^c^	48.3 ^bc^	<0.001	9.6 ^bd^	10.7 ^ce^	45.8 ^bc^	55.6 ^de^	<0.001
Vitamin B_2_ (mg/day)	0.8 ^b^	1.4 ^c^	26.7 ^bc^	<0.001	2.3 ^bd^	2.0 ^ce^	27.1 ^bc^	13.6 ^de^	<0.001
Vitamin B_6_ (mg/day)	0.8 ^b^	0.7 ^c^	32.2 ^bc^	<0.001	4.2 ^bd^	3.0 ^ce^	47.9 ^bc^	54.2 ^de^	<0.001
Vitamin B_12_ (μg/day)	0.4 ^b^	1.8 ^c^	25.0 ^bc^	<0.001	3.1 ^bd^	3.3 ^ce^	43.8 ^bc^	31.3 ^de^	<0.001
Folate (μg/day)	57.2	57.8	71.7	0.121	67.3 ^d^	62.9 ^ce^	83.3 ^cf^	97.2 ^def^	<0.001
Vitamin C (mg/day)	17.8 ^b^	15.9 ^c^	45.0 ^bc^	<0.001	19.6	17.1	29.2	21.5	0.197
Vitamin D (μg/day)	100.0	100.0	100.0	-	100.0	100.0	100.0	100.0	-
Vitamin E (mg/day)	69.3	74.4	78.3	0.270	77.7 ^d^	76.3 ^e^	91.7	94.9 ^de^	<0.001

The figures for potassium are percentages below the adequate intake (AI) level, as EAR is not available for this nutrient. Nutrient intakes are adjusted for within-subject and between subject variations by the NRC method. Subjects underreporting energy intake were excluded from the analyses. ^‡^ Derived from the Pearson Chi-square test. Superscript letters denote significant differences (*p* < 0.05) in percentages between age groups using the two-sample *Z*-test for proportions. Figures sharing the same superscript letter differ significantly from each other: ^b^ 9–10 years old *vs.* 40–60 years old; ^c^ 11–13 years old *vs.* 40–60 years old; ^d^ 9–10 years old *vs.* 50–75 years old; ^e^ 11–13 years old *vs.* 50–75 years old; ^f^ 40–60 years old *vs.* 50–75 years old.

**Table 5:** Percentages of children, middle-aged adults and postmenopausal women in Greece with usual dietary intakes below EAR, presented by age group and educational (parental in the case of children) level.

Micronutrients	Children								Middle-Aged Adults				Postmenopausal Women			
	9–10 Years Old				11–13 Years Old				40–60 Years Old				50–75 Years Old			
	≤9 Years (*N* = 72)	9–14 Years (*N* = 302)	>14 Years (*N* = 150)	*p*-Value ^‡^	≤9 Years (*N* = 95)	9–14 Years (*N* = 362)	>14 Years (*N* = 119)	*p*-Value ^‡^	≤9 Years (*N* = 34)	9–14 Years (*N* = 54)	>14 Years (*N* = 66)	*p*-Value ^‡^	≤9 Years (*N* = 95)	9–14 Years (*N* = 75)	>14 Years (*N* = 44)	*p*-Value ^‡^
	%	%	%		%	%	%		%	%	%		%	%	%	
Calcium (mg/day)	74.6 ^b^	58.9	50.0 ^b^	0.003	60.6 ^b^	57.1	44.9 ^b^	0.036	57.9	53.8	46.0	0.610	88.3	86.5	90.9	0.771
Copper (μg/day)	6.0	5.4	2.7	0.406	7.4	5.0	6.8	0.572	26.3	33.3	22.0	0.488	3.2	0.0	2.3	0.317
Iron (mg/day)	1.5	2.0	0.7	0.567	5.3 ^ab^	0.8 ^a^	0.8 ^b^	0.006	15.8	20.5	14.0	0.711	4.3	0.0	0.0	0.077
Magnesium (mg/day)	34.3	29.3	31.5	0.693	29.8	31.0	25.4	0.512	100.0 ^ab^	69.2 ^a^	56.0 ^b^	0.002	88.2	75.3	75.0	0.059
Potassium (g/day)	98.5	97.3	99.3	0.343	97.9	98.6	99.2	0.732	100.0	92.3	98.0	0.237	100.0	98.6	100.0	0.392
Selenium (μg/day)	53.7	55.2	51.4	0.746	62.8	57.3	54.2	0.453	15.8	7.7	8.0	0.556	7.5	2.7	4.5	0.379
Zinc (mg/day)	9.0	9.8	5.5	0.308	12.8	10.2	7.6	0.464	52.6	61.5	44.0	0.259	63.4	53.4	61.4	0.410
Vitamin A (μg/day)	37.3 ^b^	25.3	18.5 ^b^	0.013	27.7	22.4	26.3	0.472	52.6	43.6	40.0	0.640	12.8	5.4	11.4	0.266
Vitamin B_1_ (mg/day)	11.9	7.1	3.4	0.063	7.4	8.6	4.2	0.297	47.4	56.4	40.0	0.306	62.8	45.9	54.5	0.093
Vitamin B_2_ (mg/day)	6.0 ^ab^	1.0 ^a^	0.2 ^b^	0.002	3.2	1.7	0.8	0.424	31.6	25.6	26.0	0.876	19.1	8.1	9.1	0.073
Vitamin B_6_ (mg/day)	7.5 ^a^	1.3 ^a^	1.4	0.006	3.2	2.2	0.1	0.193	47.4	38.5	36.7	0.790	61.7	47.3	50.0	0.145
Vitamin B_12_ (μg/day)	4.5	1.3	0.7	0.105	4.3	2.8	0.8	0.291	36.8	35.9	30.0	0.717	29.8	32.4	31.8	0.929
Folate (μg/day)	70.1	60.6	60.3	0.317	69.1	58.4	57.6	0.141	84.2	79.5	72.0	0.499	97.9	95.9	97.7	0.733
Vitamin C (mg/day)	32.8 ^a^	14.8 ^a^	19.9	0.003	17.0	16.1	16.9	0.960	31.6	33.3	44.0	0.483	30.9 ^a^	13.5 ^a^	15.9	0.015
Vitamin D (μg/day)	100.0	100.0	100.0	-	100.0	100.0	100.0	-	100.0	100.0	100.0	-	100.0	100.0	100.0	-
Vitamin E (mg/day)	74.6	73.7	71.9	0.891	78.7	74.8	73.7	0.672	100.0	79.5	82.0	0.110	97.9	90.5	95.5	0.102

The figures for potassium are the percentages below adequate intake (AI) levels, as EAR is not available for this nutrient. Nutrient intakes are adjusted for within-subject and between subject variations by the NRC method. ^‡^ Derived from the Pearson Chi-square test. Superscript letters denote significant differences (*p* < 0.05) in percentages between groups with different educational level using the two-sample *Z*-test for proportions. Figures sharing the same superscript letter differ significantly from each other: ^a^ <9 years *vs.* 9–14 years; ^b^ <9 years *vs.* >14 years; ^c^ 9–14 years *vs.* >14 years.

## 4. Discussions

The current study examined the usual dietary intakes for 16 micronutrients among children and adults in Greece and further explored the role of age, sex and socio-economic status (SES) on these usual intakes, as well as on meeting the age- and sex-specific dietary intake recommendations. When assessing nutrient intakes in population groups, the use of average values can provide a general overview of nutrient intake levels, but cannot be used for the assessment of adequate or inadequate nutrient intakes [19]. The optimal method to assess the prevalence of the population with inadequate nutrient intakes is the EAR cut-point method [15]. However, as several EAR reference values for assessing nutrient intakes have been proposed by certain organizations, different findings regarding the prevalence of inadequate micronutrient intakes would be expected. For some of those EAR reference values, such as the ones recently collected and synthesized by the EURopean micronutrient RECommendations Aligned (EURRECA) project [20], the prevalence of inadequate nutrient intakes would probably be much lower or even higher compared to those reported by the current study. For example, if others, such as the reference values for nutrient intake in Germany, Austria and Switzerland (DACH reference values) were used [21], the percentage of children and adults with inadequate intakes would have been higher for several micronutrients than the percentages derived with the use of IOM’s reference values. Considering the above and for the sake of meaningful comparisons, the data derived from the present study were compared against other studies [15,16] also using IOM’s EAR cut-points to assess inadequacies.

The present study showed that inadequate nutrient intakes ranged from 0.4% in 9–10-year-old children regarding vitamin B_12_ intake to 100% across all age groups regarding vitamin D intake. With the exception of vitamin D, the prevalence of inadequacy for the majority of nutrients examined in the current study was found to increase with age in both sexes, being significantly higher in middle-aged adults and/or postmenopausal women compared to children. However, the increasing trend in the prevalence of inadequate micronutrient intakes observed with age in the present study was opposite of the trend observed for the average intakes in the case of many of these micronutrients. This is mainly attributed to the higher EAR cut-points provided by IOM for adults compared to children and adolescents and highlights once more the inappropriateness of average values for the assessment of the adequacy or inadequacy of nutrient intakes [19].

The significant age-related increases observed in the current study for several micronutrients in both sexes are consistent with previous studies conducted with children and adults in Greece [18,22,23], as well as in other developed countries [18,21]. Specifically, the current study showed the highest prevalence rates of inadequacy (*i.e.*, >80%) in postmenopausal women for the intakes of calcium, magnesium and folate. Regarding calcium, the prevalence of inadequate intakes reported by the present study is higher or comparable with the prevalence rates reported for women older than 64 years of age in other European countries, which ranged from 59.3% to 100% [18]. The gradual reduction occurring with age in the consumption of dairy products, *i.e.*, the most important food source of dietary calcium, could be one of the main reasons explaining the corresponding decrease in dietary calcium intake [24]. Regarding magnesium, dietary surveys in the United States have consistently shown that the intakes of magnesium from food sources for the majority of Americans of all ages, especially the elderly, is below EAR [25]. However, when supplements were taken into consideration, magnesium intakes were substantially improved, reaching levels well above EAR [25]. The very low use of supplements by the population living in southern Europe, including Greece (~5%) [26], may provide an interpretation for the findings of the present study with respect to magnesium intake. Furthermore, the gradual decrease observed over the last few decades in Greece in the adherence to the traditional Mediterranean diet, which is rich in foods providing sufficient magnesium, such as green leafy vegetables, fruits, nuts and whole grains, could set the basis for another interpretation of this finding [27,28]. Regarding folate, the prevalence of inadequate intake observed in the current study is much higher than the prevalence reported for other European countries, where it was found to range from 19.4% to 91% in 19–64-year-old women and from 17.6% to 45.9% in women older than 64 years [18]. Fortification of flour with folic acid, which is optional in certain European countries [29] and mandatory in the United States and Canada [30], could probably explain the lower prevalence of folate inadequate intakes in these countries compared to the prevalence observed in the current study.

The prevalence of inadequacies for the intakes of zinc and vitamins B_1_, B_2_, B_6_, B_12_, A and C were also considerably higher (*i.e*., rates ranging from 21.5% to 60%) in middle-aged men and/or women and/or postmenopausal women compared to children. Regarding, zinc the findings of the current study are in line with those reported by NHANES III, which showed that 35%–45% of adults aged 60 years or older had zinc intakes below EAR [31]. Regarding intakes of vitamins B_2_ and B_12_, the age-related decrease observed by the present study could be linked to the relevant decrease in the consumption of dairy products also occurring with age [32]. The adequacy in the intake of vitamin B_12_ by children in the HGS is further supported by the very low percentage of study participants with low vitamin B_12_ plasma levels reaching only 1.3% of the total sample (unpublished data). In confirmation of the above, the current study has shown a decrease in the average daily milk consumption from three portions in children to 1–2 portions in adults. As milk is usually identified as central in several dietary patterns also comprising food items from other basic foods groups, such as starch, fruits and vegetables [33,34,35], its lower consumption as people get older is also accompanied by a decrease in the consumption of other rich food sources of the B-complex vitamins, thus providing a possible interpretation also for the higher prevalence of inadequate intakes of vitamins B_1_ and B_6_ for adults compared to children.

Regarding intakes of vitamins A and C, the higher prevalence of inadequacies observed for 40–60-year-old men compared to 9–13-year-old boys is comparable or slightly higher than the percentages reported for adult men across Europe [18] and in the United States [36]. Although foods that are listed in the top food sources of vitamin A (*i.e.*, milk and milk desserts, ready-to-eat cereals and cheese) and vitamin C (*i.e*., fruit juice, fruits and ready-to-eat cereals) are popular among children and adolescents [37], their lower consumption by adult men could explain the age-related trends observed in the present study for males. In contrast, the very low prevalence of suboptimal vitamin A intake reported in the current study for postmenopausal women is unprecedented and requires further investigation. The same also applies for the significantly higher prevalence of suboptimal selenium intake reported for children compared to adults in both sexes.

Regarding the role of socio-economic status (SES) in the prevalence of inadequacies in micronutrient intakes, the present study examined differences among levels of education (parental in the case of children), which is one of the most reliable indices for assessing SES [38]. Although the prevalence of inadequate intakes was not found to significantly differ among SES groups for the majority of micronutrients, still a clear negative trend was observed in the case of calcium in children and magnesium in adults. The higher prevalence of inadequate calcium intake observed in the present study for children of lower SES could be due to the lower consumption of milk and ready-to-eat cereals at breakfast reported in previous studies by children and adolescents of lower SES compared to their more affluent peers [39]. In adults, a recent study showed that higher magnesium intake was associated with higher diet costs [40], thus providing an interpretation for the higher prevalence of inadequacy observed in the current study for lower SES groups. As similar higher diet costs also apply for other micronutrients, such as vitamins A and C, this could further support the higher percentages of inadequacies also reported for these two nutrients in lower compared to higher SES children and adults.

Contrary to the age- and SES-related trends reported for several micronutrients in the present study, no such trend was observed for vitamin D, as the prevalence of inadequacy was 100% across all age and SES groups. This is also supported by vitamin D status data observed for women participating in the Postmenopausal Health Study and for children in the HGS, thus strengthening this specific finding, since blood level data provide a much more reliable source of information compared to nutrient intake data. More specifically, the Postmenopausal Health Study showed that the percentage of vitamin D insufficiency (*i.e.*, serum 25(OH) vitamin D < 20 ng/mL) in postmenopausal women reached 60% during the winter period (*i.e.*, December through March) [41], while the relevant prevalence during winter months for children participating in the HGS reached 37.5% (unpublished data). Considering that the natural foods sources of vitamin D are very limited (e.g., fatty fish, cod liver oil, egg yolks), the lack of a mandatory fortification policy in Greece is probably the major diet-related factor for explaining this notable result of the present study. This finding is consistent with what has been recently reported for adults living in other Southern European counties [18]. However the prevalence of inadequate vitamin D intake seems to be considerably lower for adults and children living in Nordic countries [18], thus supporting a North-South effect in the prevalence of suboptimal vitamin D intake in Europe. Mandatory vitamin D food fortification, as well as the much higher use of supplements by populations living in Northern compared to Southern European countries (40% *vs.* 5%) [26] could set the basis not only for interpreting the results of the current and previous studies [42] on vitamin D, but also for facilitating future research and, possibly, discussions on vitamin D food fortification policy in Europe.

The findings of the current study should be interpreted in light of their strengths and limitations. Adjustments for the effects of day-to-day (within-subject) and subject-by-subject (between-subject) variability to estimate usual intakes, as well as exclusion of study participants under-reporting their food consumption could be considered as the strongest components of the methodological approach followed in the present study. Furthermore, the representativeness of the sample in the HGS and the use of the same methods and procedures to record dietary intake in all studies are additional strong points of the present work. In this context, in all three studies, dietary intake was recorded via three 24-h recalls that were conducted with study participants by rigorously trained dietitians that followed the same procedures. In contrast, the sampling methods targeting specific population groups (*i.e.*, osteopenic postmenopausal women in the PHS II and hypercholesterolemic adults in the CardioHealth Study), the small sample sizes (especially in the CardioHealth study) and the possible recruitment of more health-conscious volunteers in the case of the two adult studies may have negatively affected their representativeness, and this could be listed as one of their main limitations. Further with regard to the above, the results of the current study regarding the prevalence of inadequacies may have also been affected, since sampling of osteopenic postmenopausal women and hypercholesterolemic men and women could have possibly increased nutrient inadequacies in some study participants, due to their adherence to specific therapeutic diets (e.g., low fat diets), while the increased health-consciousness of others may have resulted in decreased nutrient inadequacies stemming from the intake of dietary supplements and/or fortified foods. Nonetheless, as the results reported in the two adult studies were broadly similar to the ones reported in the HGS, this provides an indication that the findings of the two adult studies should be considered valid enough. Another limitation of the present study is that self-reporting of food intake data via of the 24-h recall records introduces bias (e.g., such as misestimation of the exact amount of dietary intake, misreporting of food intake due to social desirability, *etc.*) to the dietary intake data derived from all studies, despite the fact that under-reporters were excluded from all studies. Furthermore, the use of the EAR cut-points to assess the prevalence of inadequate nutrient intakes in population groups is not the optimal one, as it is subjected to certain pitfalls, mainly related to the size of the study sample, the number of days for which food intake was recorded and the method used to assess dietary intake [43], which should be taken into account when interpreting such data. Lastly, the weakness of the food composition tables in providing the actual nutrient composition of all individual food items consumed by the study participants should also be listed among the methodological limitations of the current study in assessing the actual dietary intake. However, the present study followed all indicated steps to reduce, as much as possible, the aforementioned bias, such as the use of Greek food composition tables and the amendment of the diet analysis software with the nutrient content of commercially available food items consumed by children and adults in Greece.

## 5. Conclusions

In conclusion, the current study reported a wide variation in the intakes of vitamins and minerals across sex, age and SES groups. Furthermore, the highest prevalences of inadequate intakes for both sexes and all age and SES groups were observed for calcium, folate and vitamins D and E. Still, the prevalence of intakes below EAR for the vast majority of vitamins and minerals were found to be more pronounced in adults compared to children, while for some nutrients, significantly higher prevalences for inadequate intakes were observed for the lower compared to the higher SES group. These findings could provide some first guidance for the public health policy makers both in the direction of encouraging and supporting the consumption of certain food groups, as well as for considering food fortification. However, further studies are needed to support and confirm these findings.

## Supplementary Files

Supplementary File 1
