# Supplementary material for: Micronutrient Intakes among Children and Adults in Greece: The Role of Age, Sex and Socio-Economic Status
Source: Nutrients. 2014 Oct 3;6(10):4073–92. doi: 10.3390/nu6104073 (PMC4210908; doi:10.3390/nu6104073)
Supplement: Supplementary File 1 [file nutrients-06-04073-s001.docx]

**Supplementary Information**

**Table S1.** Age and sex-specific EAR cut-points [1,2] for assessing nutrients intake adequacy in groups.

| **Micronutrients** | **9–10 Years** | | **11–13 Years** | | **40–60 Years** | | **50–75 Years** |
| --- | --- | --- | --- | --- | --- | --- | --- |
|  | **Male** | **Female** | **Male** | **Female** | **Male** | **Female** | **Female** |
| Calcium (mg/day) | 1100 | 1100 | 1100 | 1100 | 800 | 31–50 years: 800/51–70 years: 1000 | 1000 |
| Copper (μg/day) | 540 | 540 | 540 | 540 | 700 | 700 | 700 |
| Iron (mg/day) | 5.9 | 5.7 | 5.9 | 5.7 | 6 | 31–50 years: 8.1/51–70 years: 5 | 5 |
| Magnesium (mg/day) | 200 | 200 | 200 | 200 | 350 | 265 | 265 |
| Potassium (g/day) * | 4.5 | 4.5 | 4.5 | 4.5 | 4.7 | 4.7 | 4.7 |
| Selenium (μg/day) | 35 | 35 | 35 | 35 | 45 | 45 | 45 |
| Zinc (mg/day) | 7 | 7 | 7 | 7 | 9.4 | 6.8 | 6.8 |
| Vitamin A (μg/day) | 445 | 420 | 445 | 420 | 625 | 500 | 500 |
| Vitamin B_1_ (mg/day) | 0.7 | 0.7 | 0.7 | 0.7 | 1.0 | 0.9 | 0.9 |
| Vitamin B_2_ (mg/day) | 0.8 | 0.8 | 0.8 | 0.8 | 1.1 | 0.9 | 0.9 |
| Vitamin B_6_ (mg/day) | 0.8 | 0.8 | 0.8 | 0.8 | 31–50 years: 1.1/51–70 years: 1.4 | 31–50 years: 1.1/51–70 years: 1.3 | 1.3 |
| Vitamin B_12_ (μg/day) | 1.5 | 1.5 | 1.5 | 1.5 | 2.0 | 2.0 | 2.0 |
| Folate (mg/day) | 250 | 250 | 250 | 250 | 320 | 320 | 320 |
| Vitamin C (mg/day) | 39 | 39 | 39 | 39 | 75 | 60 | 60 |
| Vitamin D (μg/day) | 10 | 10 | 10 | 10 | 10 | 10 | 10 |
| Vitamin E (mg/day) | 9 | 9 | 9 | 9 | 12 | 12 | 12 |

* The cut-points provided for Potassium correspond to Adequate Intakes (AI) as EARs are not available for this specific nutrient.

**References**

1. Institute of Medicine. *Dietary Reference Intakes. Applications in Dietary Assessment*; National Academies Press: Washington, DC, USA, 2000.

2. Ross, A.C. The 2011 report on dietary reference intakes for calcium and vitamin D. *Public Health Nutr.* **2011**, *14*, 938–939.
